# A new player in interstitial cystitis/bladder pain syndrome: platelet‐activating factor – PAF and its connection to smoking

**DOI:** 10.14814/phy2.13235

**Published:** 2017-04-13

**Authors:** Florenta Aura Kullmann

**Affiliations:** ^1^ Department of MedicineUniversity of Pittsburgh School of MedicinePittsburghPennsylvania15261

Interstitial cystitis/bladder pain syndrome (IC/BPS) is a debilitating chronic condition characterized by suprapubic pain, urinary frequency and urgency. The etiology is complex and not well understood and treatments are not satisfactory (Hanno et al. 2011). Bladder examination revealed a number of anomalies in the urothelium, including petechial hemorrhages (glomerulations), ulcers (Hunner's ulcers), denudation, tears and thinning (Hanno et al. 2011; Keay et al. 2014). Functional studies revealed a number of alterations in urothelial receptors/ion channel function (e.g., purinergic, transient receptor potential channels) and transmitter release (e.g., ATP, NO) from the urothelium in response to mechanical or chemical stimuli (Hanno et al. 2011; Keay et al. 2014). Although all of these changes are likely to contribute to pain and urinary symptoms, precise mechanisms are unclear. Life style factors, including diet, exercise, alcohol consumption and smoking, significantly impact IC/BPS symptoms. Smoking is associated with worsening of symptoms (Kennedy et al. 2006; Song et al. 2009; Tettamanti et al. 2011; Mobley and Baum 2015), however, the mechanisms underlying this effect are not understood.

The manuscript “Cigarette Smoke‐Induced Urothelial Cell Damage: Potential Role of Platelet‐Activating Factor” by Kispert et al., identifies a pathway by which smoking could lead to alterations in urothelial cells consistent with those observed in the bladders of IC/BPS patients. Moreover, it raises the possibility that this pathway is dysregulated in the urothelium of IC/BPS patients, regardless of smoking status, thus providing opportunities for new therapies. The authors focus on platelet‐activating factor (PAF), a potent proinflammatory mediator produced and released by a variety of cells including epithelial cells. PAF acts through a G‐protein coupled receptor (PAFR) and triggers immune system responses encompassing activation of leukocytes, production of reactive oxidative species, and increases in inflammatory cytokines (Il‐6, TNF), iNOS and COX‐2 (Yost et al. 2010). Consequently, PAF has been implicated in a number of pathologies associated with chronic inflammation, including asthma, rheumatoid arthritis, inflammatory bowel diseases, and others (Nassif et al. 1996; Kasperska‐Zajac et al. 2008; Yost et al. 2010). Although PAF has been widely implicated in chronic inflammatory diseases, its role in IC/BPS is relatively unexplored.

This study uses a combination of in vitro and in vivo techniques to provide evidence for a role of PAF in IC/BPS and to demonstrate a relationship between smoking, PAF and urothelial histopathology in IC/BPS. The authors establish that primary human urothelial cells (HUC) and immortalized urothelial cells from healthy controls (UT‐C) and IC/BPS patients (UT‐IC), express a key enzyme necessary for PAF synthesis, iPLA_2_ *β*. Importantly, the activity of this enzyme was increased in cells derived from IC/BPS patients compared to controls. Exposure of cultured HUC, UT‐C and UT‐IC cells to cigarette smoking extract (CSE) resulted in iPLA_2_ *β*‐mediated PAF production, which was higher in UT‐IC cells. Moreover, urinary levels of PAF were increased in IC/BPS patients, and further elevated in IC/BPS smokers, compared to healthy controls. Together, these findings indicate that PAF is produced and released by urothelial cells and its production is increased in IC/BPS and further augmented by exposure to smoke.

A hallmark of urothelial dysfunction in IC/BPS is decreased ability to repair the urothelial barrier after injury, due to impaired cell proliferation (Keay et al. 2003, 2014). Moreover, wound healing is slower in smokers versus nonsmokers (McDaniel and Browning 2014). Consistent with impaired repair, this study shows slower re‐epithelization rates in UC‐IC than in UC‐C cells in a wound healing assay. CSE reduced the re‐epithelization rates and (S)‐ Bromoenol lactone ((S)‐BEL), an inhibitor of the iPLA_2_ *β* activity, partially rescued the recovery rates in all cell types. Together, these experiments suggest that PAF could be involved in urothelial repair after injury and manipulating PAF levels (decreasing with (S)‐BEL or increasing with CSE) directly modulates the repair, possibly impacting cell proliferation.

Finally the authors exposed wild‐type mice and mice lacking the iPLA_2_ *β* enzyme (iPLA_2_ *β* ^−/−^) to smoke for 6 months. Histological evaluation of the bladder revealed denudation and thinning of the urothelium, and results from a previous study indicated infiltration of inflammatory cells into the bladder wall (Marentette et al. 2015), in wild‐type mice but not in iPLA_2_ *β* ^−/−^ mice. Interestingly, the expression of PAF and its receptor was elevated in the urothelium of the wild type mice but not of the iPLA_2_ *β* ^−/−^ mice exposed to smoke. These in vivo data provide a link between exposure to smoke, PAF‐mediated signaling and abnormal urothelial histology.

In summary, the results demonstrate that PAF signaling pathways are upregulated in IC/BPS and that exposure to smoke cause further upregulation. Exogenous cigarette smoking extract in urothelial cells and chronic smoke exposure in mice increased PAF production and PAFR expression in urothelial cells, reduced wound healing rates, and produced histological alterations in the urothelium consistent with those observed in the bladders of IC/BPS patients.

The results raise several interesting questions and open several avenues that could be further explored:

1. Does the PAF signaling pathway play a role in the development and/or progression of urothelial dysfunction in IC/BPS, regardless of the smoking status? To address this question, studies using animal models of cystitis are needed. While there is an understanding that no animal model faithfully reproduces the human condition, animal models, and especially the use of iPLA_2_ *β* ^−/−^ mice (and other genetically engineered lines), coupled with pharmacological manipulations, provide ways to assess the role of PAF pathways in cystitis, which can lead to potential targets for intervention. Additionally, in vitro models of urothelial cell cultures treated with PAF and PAF antagonists can be further used to gain insights into the role of PAF in altered functional properties of urothelial cells in IC/BPS.
2. If PAF signaling is altered in IC/BPS, can antagonists of PAFR or inhibition of PAF production prevent and/or improve urothelial dysfunction and ultimately improve IC/BPS symptoms? Furthermore, does inhibition of PAF pathways help managing cigarette smoke‐induced urothelial damage, thus alleviating smoking‐exacerbated IC/BPS symptoms? While early clinical trials did not demonstrate efficacy of PAFR antagonists in diseases such as septic shock, asthma, and pancreatitis (Vincent et al. 2000), there is renewed interest in different treatment approaches, such as local delivery of compounds into the bladder. As PAF is likely not the only pathway altered in IC/BPS, targeting PAF may be used in conjunction with existing treatments. For example, treatments aimed at restoring the glycosaminoglycan (GAG) layer to protect the urothelium and prevent infiltration of harmful substances into the bladder wall, show moderate benefits in patients. Would a combination of inhibitors of PAF signaling pathways and GAG replenishing compounds be more effective as it may decrease the inflammation while aiding restoration of the urothelium?
3. Finally, can PAF and/or associated pathways be used as biomarkers for diagnosis of IC/BPS and/or for determining whether a specific treatment has a positive effect?

In summary, this manuscript highlights PAF and its associated signaling as novel players in urothelial alterations reported in IC/BPS patients, shines light on a possible mechanism underlying smoking‐exacerbated IC/BPS symptoms, and provides potential therapeutic targets to be further explored.
